# Evaluating State Space Discovery by Persistent Cohomology in the Spatial Representation System

**DOI:** 10.3389/fncom.2021.616748

**Published:** 2021-04-08

**Authors:** Louis Kang, Boyan Xu, Dmitriy Morozov

**Affiliations:** ^1^Redwood Center for Theoretical Neuroscience, University of California, Berkeley, Berkeley, CA, United States; ^2^Neural Circuits and Computations Unit, RIKEN Center for Brain Science, Wako, Japan; ^3^Department of Mathematics, University of California, Berkeley, Berkeley, CA, United States; ^4^Computational Research Division, Lawrence Berkeley National Laboratory, Berkeley, CA, United States

**Keywords:** topological data analysis, neural manifold, dimensionality reduction, neural decoding, spatial representation, grid cells, medial entorhinal cortex

## Abstract

Persistent cohomology is a powerful technique for discovering topological structure in data. Strategies for its use in neuroscience are still undergoing development. We comprehensively and rigorously assess its performance in simulated neural recordings of the brain's spatial representation system. Grid, head direction, and conjunctive cell populations each span low-dimensional topological structures embedded in high-dimensional neural activity space. We evaluate the ability for persistent cohomology to discover these structures for different dataset dimensions, variations in spatial tuning, and forms of noise. We quantify its ability to decode simulated animal trajectories contained within these topological structures. We also identify regimes under which mixtures of populations form product topologies that can be detected. Our results reveal how dataset parameters affect the success of topological discovery and suggest principles for applying persistent cohomology, as well as persistent homology, to experimental neural recordings.

## 1. Introduction

The enormous number of neurons that constitute brain circuits must coordinate their firing to operate effectively. This organization often constrains neural activity to low-dimensional manifolds, which are embedded in the high-dimensional phase space of all possible activity patterns (Gao and Ganguli, 2015; Gallego et al., 2017; Jazayeri and Afraz, 2017; Saxena and Cunningham, 2019). In certain cases, these low-dimensional manifolds exhibit non-trivial topological structure (Curto, 2016). This structure may be imposed externally by inputs that are periodic in nature, such as the orientation of a visual stimulus or the direction of an animal's head. It may also be generated internally by the network itself; for example, the grid cell network constructs periodic representations of physical space which outperform non-periodic representations in several ways (Fiete et al., 2008; Sreenivasan and Fiete, 2011; Mathis et al., 2012; Stemmler et al., 2015; Wei et al., 2015; Sanzeni et al., 2016; Mosheiff et al., 2017). In either case, detecting and interpreting topological structure in neural data would provide insight into how the brain encodes information and performs computations.

One promising method for discovering topological features in data is persistent cohomology (Edelsbrunner and Harer, 2010; de Silva et al., 2011; Edelsbrunner and Morozov, 2017). By tracking how the shape of the data changes as we examine it across different scales—thickening data points by growing balls around them—persistent cohomology detects prominent topological features in the data, such as loops and voids. This knowledge helps to identify the low-dimensional manifolds sampled by the data, and in particular to distinguish between tori of different intrinsic dimensions. Furthermore, it enables parametrization of the data and navigation of the underlying manifolds.

**Figure 1:**
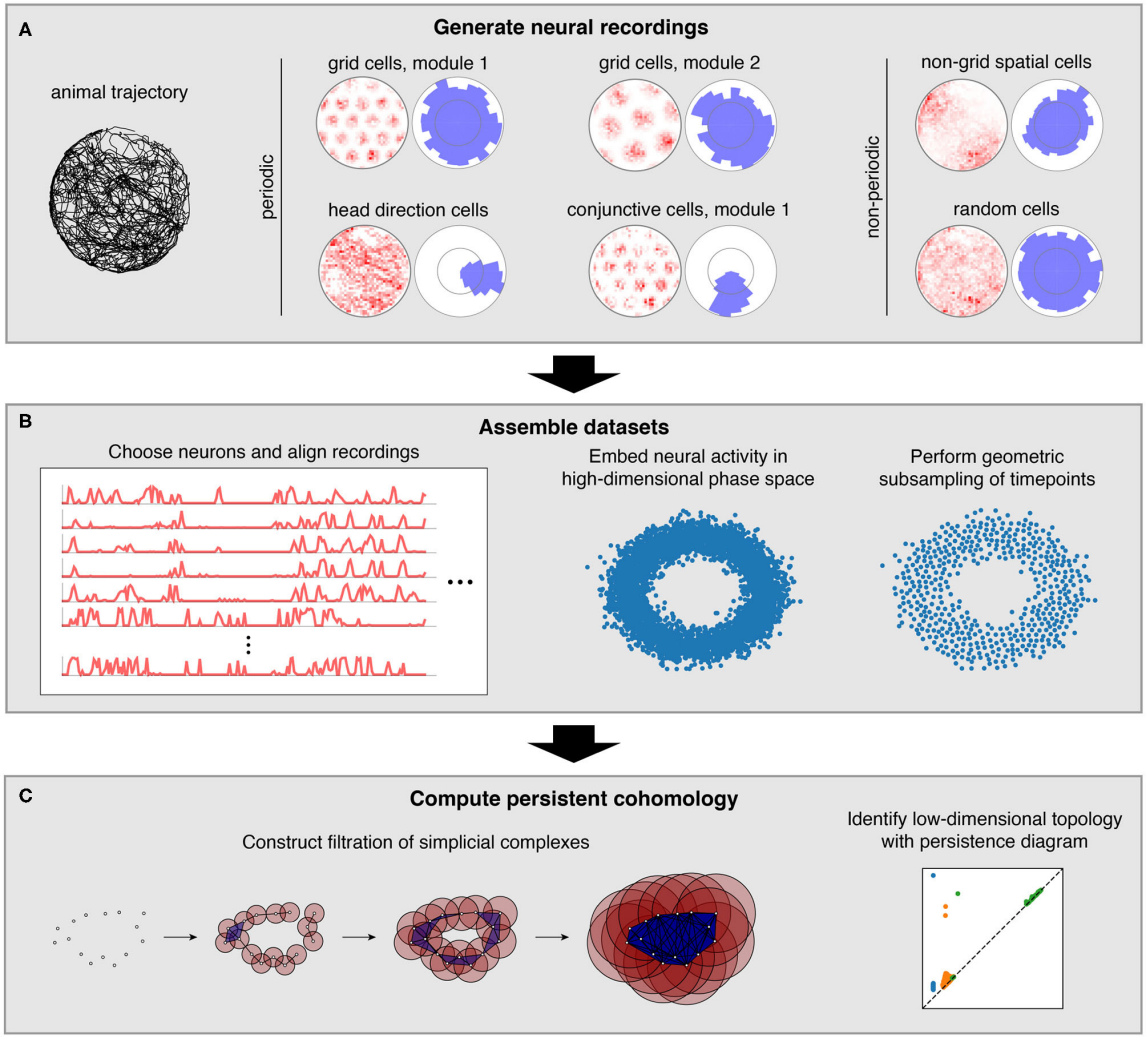
Pipeline for simulations and data analysis. **(A)** We generate activities for multiple neural populations along an experimentally recorded rat trajectory. For each population, we plot activity maps as a function of position (left) and direction (right) for one example neuron. **(B)** Then we choose neurons for topological analysis and form a high-dimensional vector of their firing rates at each timepoint along the trajectory. For computational tractability, we eliminate the most redundant points using a geometric subsampling algorithm. **(C)** We compute persistent cohomology on these subsampled timepoints to identify low-dimensional topological structure.

We characterize how persistent cohomology can discover topological structure in neural data through simulations of the brain's spatial representation system. This system contains several neural populations whose activity exhibits non-trivial topology, which we term *periodic neural populations* (Figure 1A). Grid cells fire when an animal reaches certain locations in its environment that form a triangular lattice in space (Hafting et al., 2005). In each animal, grid cells are partitioned into 4–10 modules (Stensola et al., 2012). Within each module, grid cells share the same scale and orientation but their lattices have different spatial offsets. Modules appear to increase in scale by a constant ratio and exhibit differences in orientation (Stensola et al., 2012; Krupic et al., 2015). Head direction cells fire when an animal's head is oriented in a certain direction relative to its environment (Taube et al., 1990). They respond independently of the animal's position. Finally, conjunctive grid × head direction cells respond when an animal is located at the vertices of a triangular lattice and is oriented in a certain direction (Sargolini et al., 2006). Like grid cells, conjunctive cells are also believed to be partitioned into modules.

We also consider neural populations whose activity exhibits trivial topology, which we will term *non-periodic neural populations* (Figure 1A). Place and non-grid spatial cells are part of the spatial representation system, and they fire in one or multiple regions of the environment (O'Keefe and Dostrovsky, 1971; Diehl et al., 2017; Hardcastle et al., 2017). These two populations are found in different brain regions, and the former tend to have sharper spatial selectivity compared to the latter. Finally, we simulate neurons with irregular activity that exhibits no spatial tuning. We imagine these *random cells* may be responding to non-spatial stimuli or representing internal brain states.

Persistent cohomology, as well as the closely related technique persistent homology, has recently been applied to experimental neural recordings within the spatial representation system. It was used to discover topological structure (Chaudhuri et al., 2019; Rybakken et al., 2019) and decode behavioral variables (Rybakken et al., 2019) from head direction cells. It was also used to do the same for grid cell recordings (Gardner et al., 2021), and researchers have demonstrated topological discovery in simulated grid cell data (Chaudhuri et al., 2019). These works have improved our understanding of the large-scale organization of spatial representation circuits through persistent cohomology.

In contrast to the research described above, we aim to comprehensively explore the capabilities of persistent cohomology for simulated datasets. With complete control over the data, we can identify features that improve topological discovery and features that disrupt it. We can also freely generate datasets with varied quantities and proportions of different neural populations. A greater number of neurons embeds underlying activity manifolds in higher dimensions, which can strengthen the signal. However, experimental limitations impose bounds to this number. Our simulations allow us to evaluate persistent cohomology in regimes currently accessible by experiments, as well as in regimes that may soon become experimentally tractable due to advances in recording technology (Jun et al., 2017).

## 2. Results

### 2.1. Overview of Methods and Persistence Diagrams

In this work, we simulate neural populations within the spatial representation system, prepare the simulated data for topological analysis, and compute persistent cohomology to discover topological structure within the data (Figure 1). We will now briefly describe each of these three stages; a complete explanation is provided in the Methods section.

To generate neural recordings, we define tuning curves as a function of position and direction. For each grid module, we first create a triangular lattice in space. Each grid cell has peaks in its positional tuning curves at a randomly chosen offset from each lattice point. Its directional tuning curve is uniform. Head direction cells have peaks in their directional tuning curves at a randomly chosen angle and have uniform positional tuning curves. Conjunctive cells have positional tuning curves like grid cells and directional tuning curves like head direction cells. We describe tuning curves for the non-periodic neural populations in the Methods section.

These tuning curves are applied to an experimentally extracted trajectory of a rat exploring its circular enclosure, producing an activity, or firing rate, for each neuron at 0.2 s intervals. This simulates the simultaneous recording of a large number of neurons from the medial entorhinal cortex and the binning of their spikes into firing rates. The time series spans 1,000 s, or 5,000 data points. Figure 1A shows examples of these time series data mapped back onto spatial coordinates. Next, we choose a subset of these neurons and pre-process it for topological data analysis (Figure 1B). We form a vector of neural activities at each timepoint, which produces a point cloud in high-dimensional phase space. We wish to subsample it for computation tractability while maintaining as much evidence of topological structure embedded within it. To do so, we use a geometric subsampling algorithm that roughly eliminates the most redundant points, reducing the 5,000 timepoints down to 1,000.

**Figure 2:**
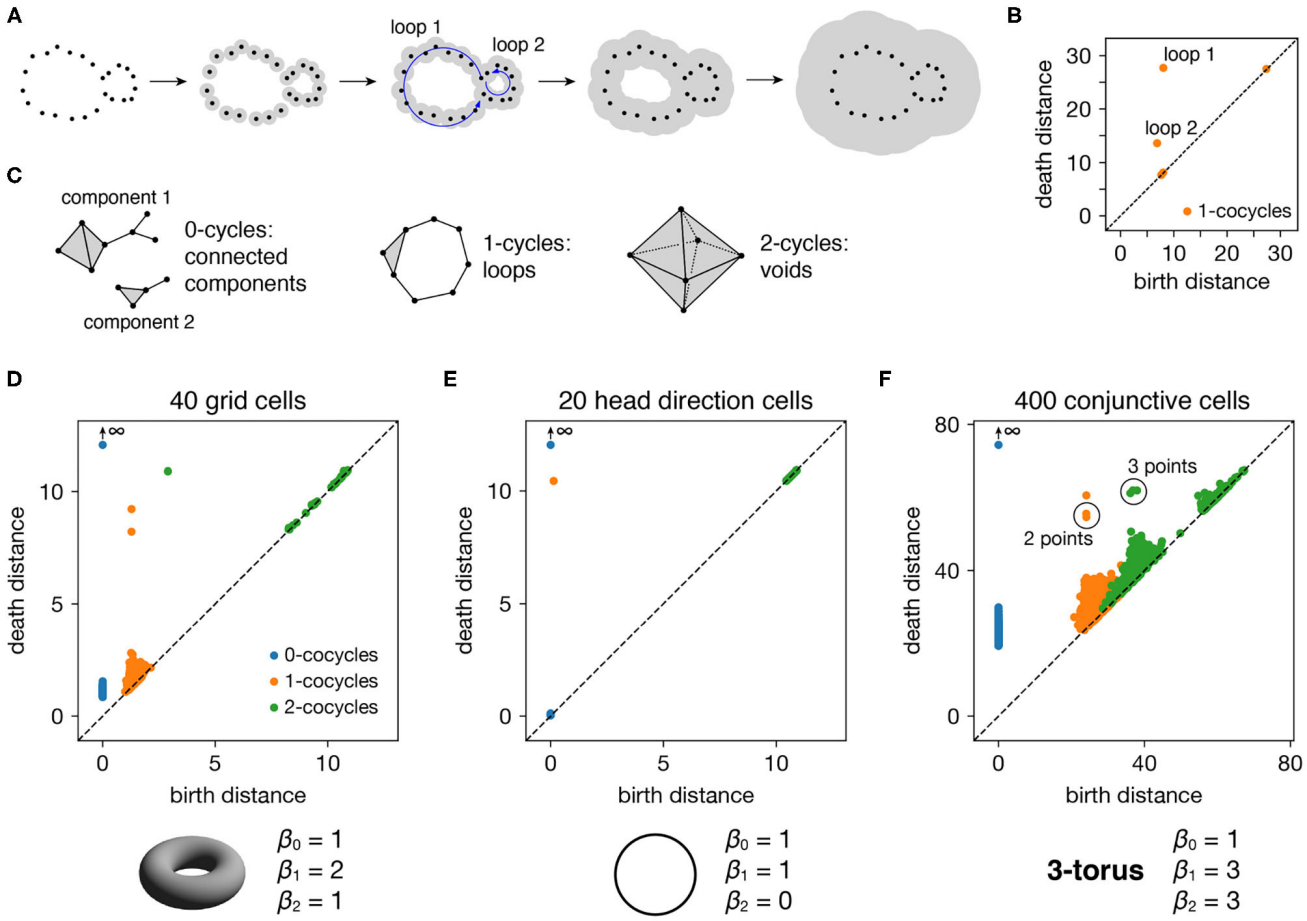
Persistence diagrams for periodic neural populations. **(A–C)** Interpreting persistence diagrams. **(A)** Persistent (co)homology involves generating a filtration of a dataset in which we connect points within ever-expanding distances. One-dimensional loops will be born and then die at various distances. **(B)** These values are plotted in a persistence diagram where each point corresponds to a single loop. The points located farthest from the diagonal are the most persistent and correspond to significant topological features. **(C)** This process can be performed for features of arbitrary dimension *k*. **(D–F)** Persistence diagrams for periodic neural populations (top) and identified topological spaces (bottom). We compare the number of persistent *k*-(co)cycles to the *k*-th Betti numbers β_*k*_ of different topological spaces to infer the underlying topological structure of the dataset. **(D)** Grid cells from module 1 exhibit one persistent 0-cocycle, two persistent 1-cocycles, and one persistent 2-cocycle, which corresponds to a torus. **(E)** Head direction cells exhibit one persistent 0-cocycle, one persistent 1-cocycle, and no persistent 2-cocycles, which corresponds to a circle. **(F)** Conjunctive cells exhibit one persistent 0-cocycle, three persistent 1-cocycles, and three persistent 2-cocycles, which corresponds to a 3-torus.

Finally, we apply persistent cohomology to this subsampled point cloud (Figure 1C). We describe this technique colloquially here, in terms of its dual, persistent homology. Both produce the same persistence diagrams, but we use cohomology throughout the paper both because it is faster to compute and because it allows us to parameterize the data. See the Methods section for a precise description. From the point cloud, we form a Vietoris–Rips filtration, which is a nested sequence of simplicial complexes. Each complex consists of all cliques in the near-neighbor graph, which contains all edges between points at distance at most *r* apart. As the threshold *r* increases, more edges enter the graph, and more cliques enter the Vietoris–Rips complex. Throughout this process, cycles (e.g., 1-dimensional loops) appear and get filled in the complex (Figure 2A). There is a unique way to pair the distance thresholds at which cycles are born and die.

All such birth and death distances are collected into a persistence diagram (Figure 2B). The points farthest from the diagonal correspond to the most persistent cycles that appear for the longest range of distance thresholds. They recover topological structure in the space sampled by the point cloud, which corresponds to the processes underlying the data—in our case, the spatial representation networks and external inputs. Persistent (co)homology is stable: the persistent points will remain in the diagram if we make small changes to the data, such as selecting slightly different timepoints or perturbing their values by a small amount of noise. The points closest to the diagonal would appear even if the processes underlying the data lack topological structure, and they are usually interpreted as noise.

The process we described above keeps track of cycles of different dimensions (Figure 2C). Besides loops (1-dimensional cycles), it tracks connected components (0-dimensional cycles), voids (2-dimensional cycles), and higher-dimensional topological features, which lack a colloquial name. The number of independent *k*-cycles is called the *k*-th Betti number and is a topological invariant of a space. We can infer the topology of a dataset by comparing the number of persistent *k*-(co)cycles to the *k*-th Betti numbers of conjectured ideal spaces, such a circle or a torus. Note that for every dataset, the 0-(co)cycle corresponding to the entire point cloud will never die, so we consider its death distance to be infinity.

### 2.2. Persistent Cohomology for Periodic Neural Populations

Each periodic neural population spans a particular topological space. We recover these relationships when we compute persistent cohomology of our simulated data (Figures 2D–F). Each grid cell is active at one location in a unit cell that is tiled over 2D space. Grid cells within a single module share the same unit cell but differ in their active location, so each grid module spans a torus, which is periodic in two directions (Curto, 2016). Similarly, head direction cells span a circle and each conjunctive cell module spans a 3-torus. The correspondence between our results and predicted topological spaces validates the basic capabilities of our methods.

**Figure 3:**
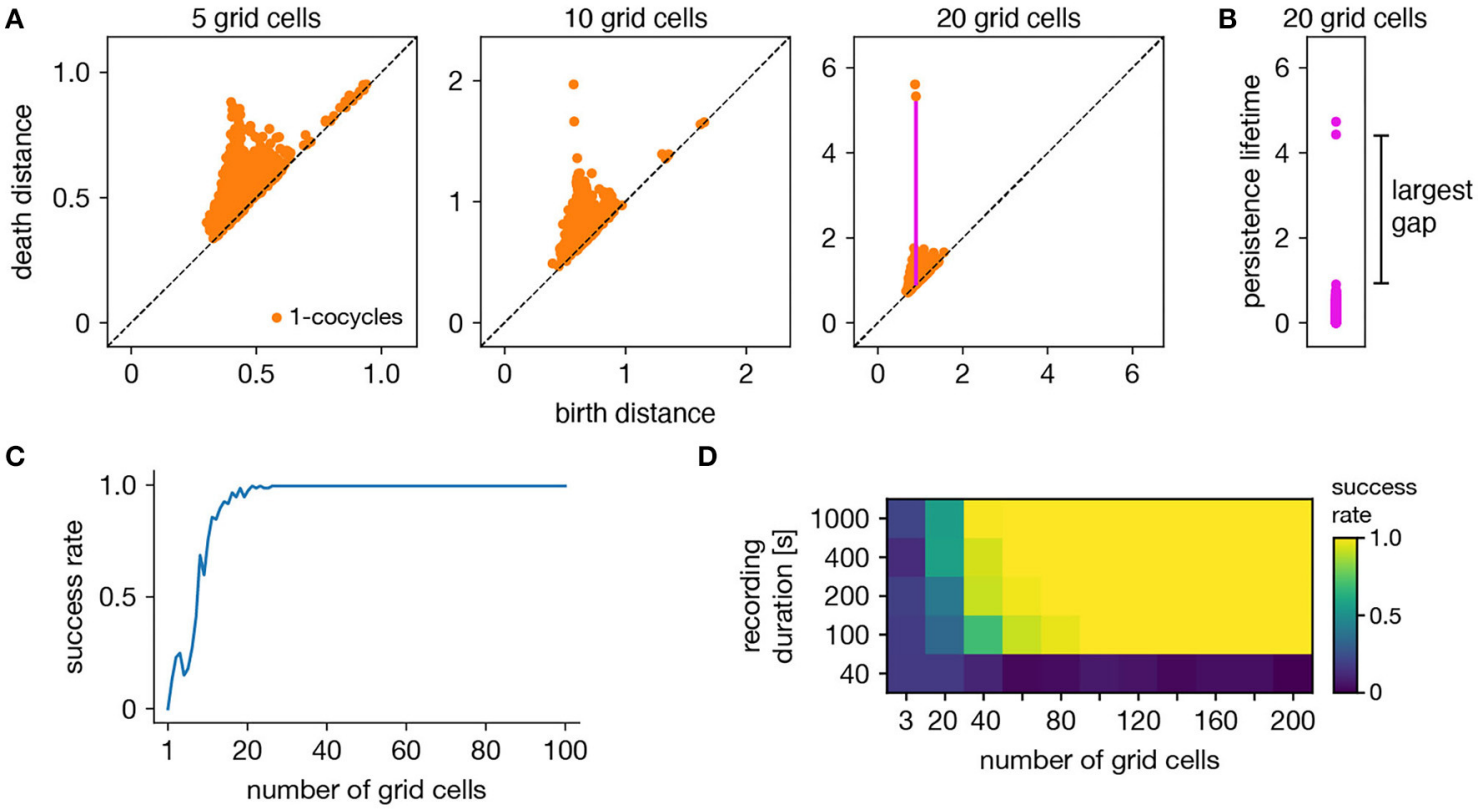
Success rates of persistent cohomology for grid cells. **(A)** As the number of grid cells increases, two persistent 1-cocycles emerge. To define persistence precisely, we consider the persistence lifetime of each 1-cocycle, which is the difference between its birth and death distances (length of the magenta line). **(B)** We identify the largest gap in persistence lifetimes and define persistent cocycles as those above this gap. **(C)** Since a grid module has a toroidal topology for which β_1_ = 2, we define success as a persistence diagram with two persistent 1-cocycles. We determine success rates by generating 100 replicate datasets. Success rate increases with the number of grid cells. **(D)** Success rates for different durations extracted from the full simulated recording. Topological discovery benefits from longer recording durations and more neurons.

The ability of persistent cohomology to discover topological structure depends on the number of neurons in the dataset, or equivalently, the dimension of the time series embedding. Using the grid cell population as an exemplar, we form multiple datasets with randomly selected neurons to measure the success rate of persistent cohomology as a function of neuron count (Figure 3A). To measure success, we only use the first cohomology group H^1^, which contains 1-cocycles. We define successful discovery of the grid cell torus as a persistence diagram with two persistent 1-cocycles, and we define what it means to be persistent precisely using the commonly used *largest-gap heuristic*. We calculate the lifetime of each cocycle, which is the difference between its death and birth and corresponds to the vertical distance to the diagonal of its point in the persistence diagram (Figures 3A,B). We find the largest gap in the lifetimes and consider points above this gap to be persistent (Figure 3B). Figure 3C shows that reliable discovery of the torus using this heuristic can be achieved with ≈20 simulated, idealized grid cells. It also shows that increasing the number of grid cells improves the success rate. This occurs because topological discovery relies on having enough grid cells such that their fields provide a sufficiently uncorrelated coverage of the unit cell. Instead of using data spanning the full 1,000 s-long trajectory, which corresponds to 5,000 timepoints, we extract portions spanning various lengths. Discovery of the torus requires enough samples of its manifold structure, which is achieved in this case starting at 100 s (Figure 3D). Thus, persistent cohomology generally thrives in the large-dataset limit with long neural recordings of many neurons.

**Figure 4:**
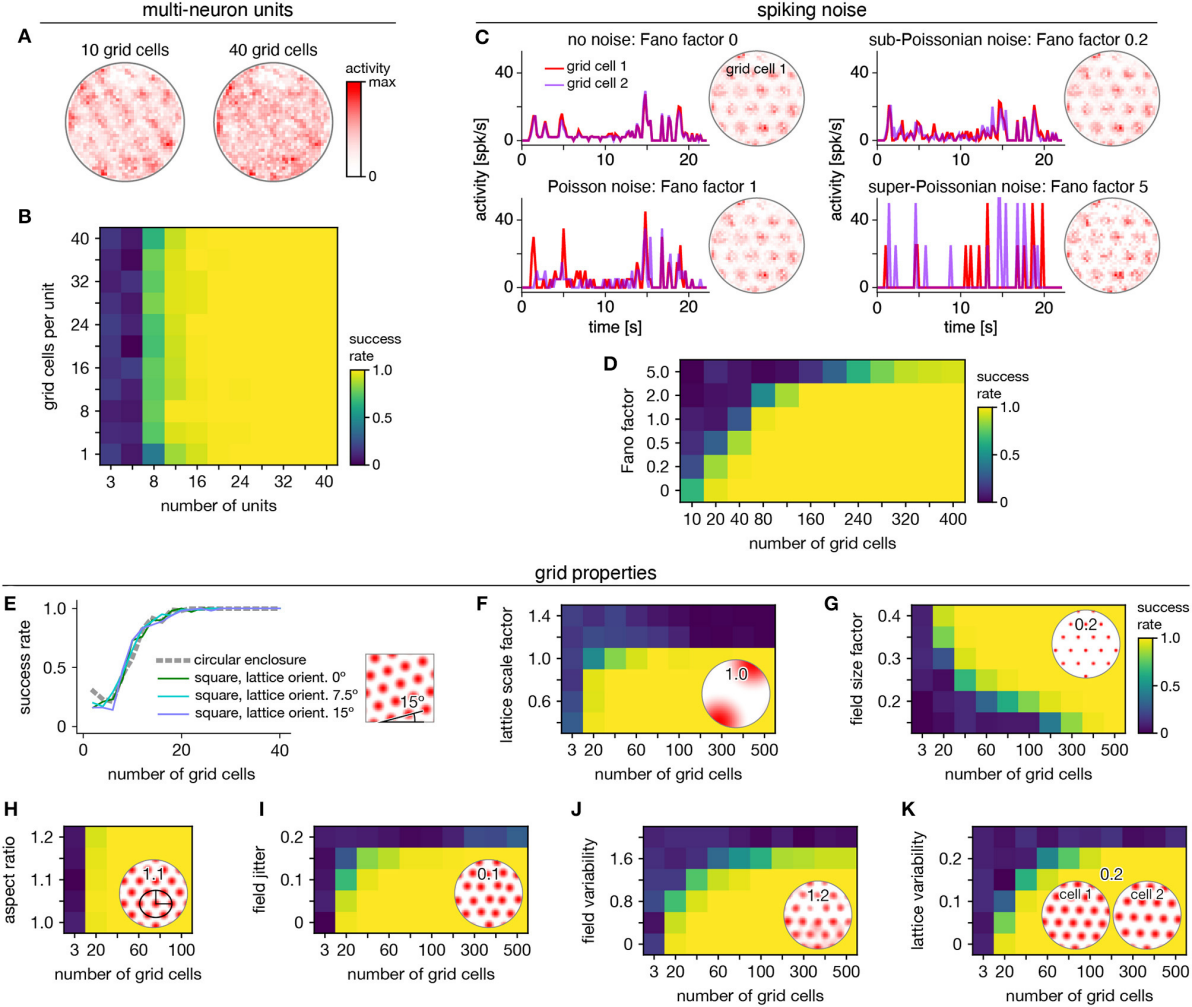
Robustness of persistent cohomology for grid cells. **(A,B)** Linearly combining activities from multiple neurons before applying persistent cohomology. **(A)** Example positional activity maps for multi-neuron units. **(B)** The success rate is largely independent of the number of neurons per multi-neuron unit. **(C,D)** Introducing Poisson-like spiking noise with different amounts of variability, as measured by the Fano factor. **(C)** For each Fano factor, time series for two grid cells with very similar offsets (left) and positional activity map of the first (right). **(D)** Persistent cohomology requires more neurons to achieve success with higher Fano factors. **(E–K)** Success rates across many different grid cell variables with insets depicting example values. See Methods section 4.1.7 for a complete description of each variable. **(E)** Introducing a square enclosure at various lattice orientations. **(F)** Changing the lattice scale factor, which is grid scale divided by enclosure diameter. **(G)** Changing the field size factor, which is grid field diameter divided by grid scale. **(H)** Stretching triangular grids to various aspect ratios. **(I)** Jittering the position of each field by random vectors multiplied by grid scale. **(J)** Introducing variability in shape and overall activity across grid fields. **(K)** Introducing variability in lattices across grid cells.

Persistent cohomology can succeed for mixed signals. Separation of raw electrode recordings into single-neuron spike trains may not always be possible or desired. To address this scenario, we form multi-neuron units by linearly combining time series of neural activity across different grid cells. The mixing coefficients are drawn from a uniform random distribution and then normalized. Example activity maps of these multi-neuron units as a function of position are shown in Figure 4A. The combination of many neurons destroys the classic responses exhibited by individual grid cells. Yet, multi-neuron units retain topological information associated with the grid module that can be recovered by persistent cohomology (Figure 4B). The success rate for discovering toroidal topology is remarkably independent of the number of grid cells in each unit. Successful recovery from randomly mixed signals is not entirely unexpected. The preservation of distances under random projections has been studied extensively in statistics [cf. Johnson–Lindenstrauss lemma (Johnson and Lindenstrauss, 1984)].

Persistent cohomology can also succeed in the presence of spiking noise. To simulate such noise, we use our generated activity as a raw firing rate that drives a Poisson-like random process (see Methods). We construct this process to have different Fano factors, which is the variance in the random process for a given firing rate divided by the firing rate. When the Fano factor is 1, the random process is Poisson. Figure 4C shows activity time series for two grid cells that have very similar tuning curves and thus very similar raw firing rates, which can be seen in the noise-free condition (top left). Higher Fano factors lead to more variability both across time for each neuron and across neurons. Persistent cohomology can still recover the toroidal topology of the grid module, though more neurons are required for higher Fano factors (Figure 4D). In the mammalian cortex, Fano factors lie around ~0.5–1.5 (Shadlen and Newsome, 1998; Burak and Fiete, 2009). Applying this regime to our simulations implies that ≈80 grid cells are required for reliable topological discovery, but we acknowledge the large differences between simulated and experimental data which may substantially increase this number.

We further test the robustness of persistent cohomology across wide ranges of properties associated with grid cells (Figures 4E–K). Using a trajectory extracted from an animal exploring a square enclosure does not meaningfully change the success rate (Figure 4E). Grid modules have been shown to favor a lattice orientation of 7.5° relative to square enclosures (Stensola et al., 2015), but this orientation does not affect topological discovery. Similarly, persistent cohomology is not strongly affected by the aspect ratio of the triangular grid (Figure 4H). Changes in grid dimensions can have a stronger effect. Persistent cohomology fails when the grid scale exceeds the size of the enclosure, which makes sense because the grid module unit cell can no longer be fully sampled (Figure 4F). When the scale does not change but the size of each firing field is decreased, more grid cells are required to produce toroidal structure in the data since each neuron covers less of the unit cell (Figure 4G). Under various forms of variability within grid cell tuning curves, the success of persistent cohomology can be maintained if more neurons are recorded, up to a degree (Figures 4I–K). Beyond critical values, however, variability appears to catastrophically disrupt topological discovery in a way that cannot be overcome with more grid cells. The extra heterogeneity conveyed by an additional neuron overwhelms its contribution to topological structure. Thus, an assessment of tuning curve variability may be a crucial component in the application of persistent cohomology to neural data.

### 2.3. Animal Trajectory Decoded Through Topological Coordinates

**Figure 5:**
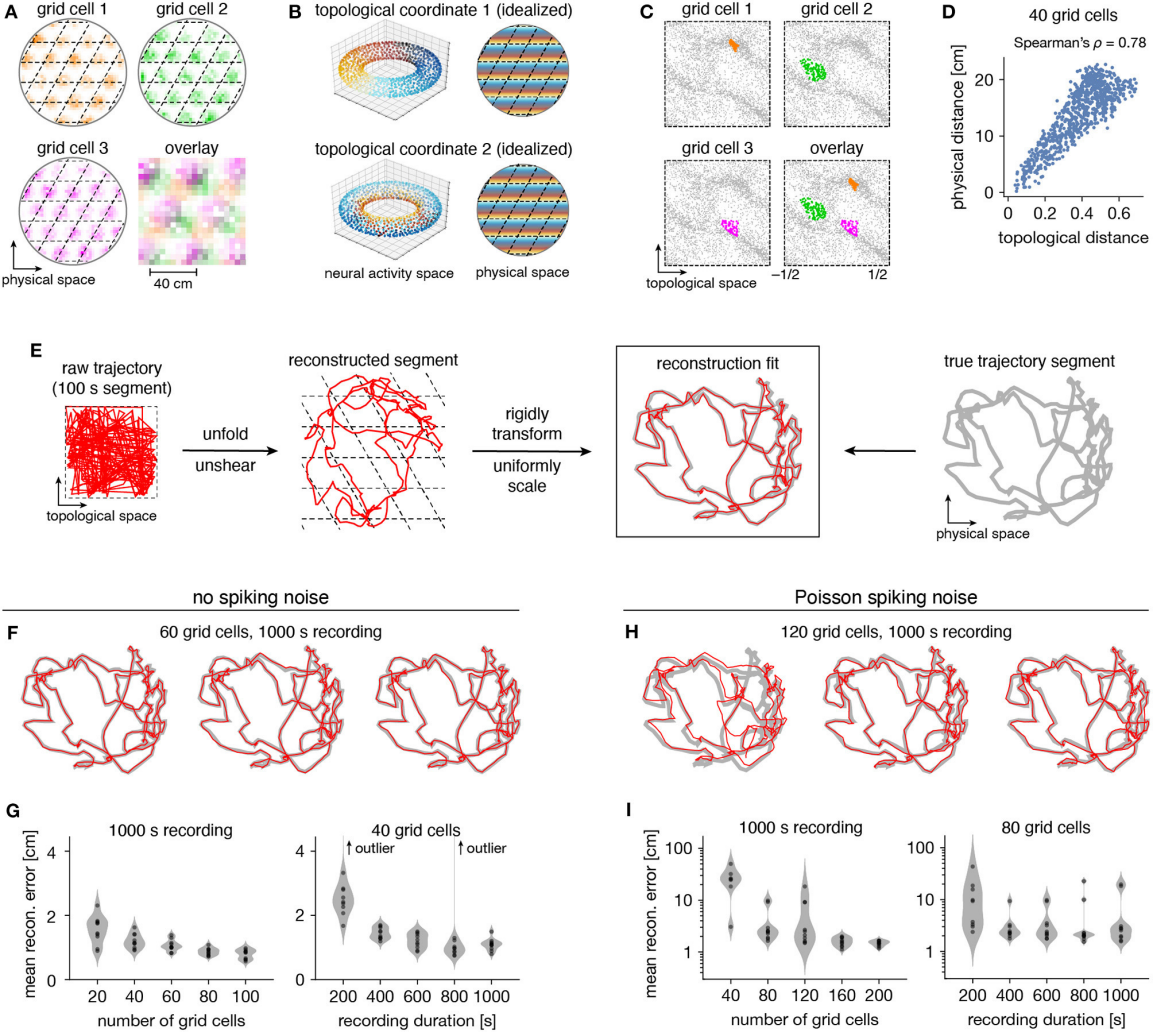
Correspondence between spatial and topological coordinates for grid cells. **(A)** Positional activity maps. A grid module tiles physical space with a rhombic unit cell (black dashed lines) with a different offset for each grid cell. **(B)** Schematic of circular coordinates (color) for the two persistent 1-cocycles of a grid module that define a topological space (left) and their mapping onto physical space (right). **(C)** Firing fields in the topological space of neural activities. All timepoints (gray) and those for which a grid cell is the most active in the population (color). **(D)** Topological distances between firing fields depicted in **(C)** are correlated with physical distances between grid offsets depicted in **(A)**. Each point represents distances between two grid cells. **(E)** Reconstructing a segment of animal trajectory in the topological space of neural activities and fitting it to the true trajectory segment. **(F,G)** Reconstructions for grid cells without spiking noise. **(F)** Sample reconstruction fits. **(G)** Mean reconstruction error is the distance between reconstructed and actual positions averaged over timepoints. For each condition, we generate 10 replicate datasets, shown as violin plots with points representing individual values. Error generally decreases with increasing grid cell number and trajectory duration. **(H,I)** Same as **(F,G)** but for grid cells generated with Poisson spiking noise and error plotted on a logarithmic scale. Geometric subsampling was not performed. Persistent cohomology can achieve low reconstruction error with large enough grid cell number and trajectory duration.

Persistent cohomology can not only discover topological structure in neural data, but it can also decode information embedded within this structure. Recall that a grid module defines a triangular lattice in physical space with fields of each grid cell offset in the rhombic unit cell (Figure 5A). The periodicities of this unit cell along the two lattice vectors are detected by persistent cohomology as two persistent 1-cocycles belonging to a torus (as seen in Figure 2D). We can assign circular coordinates (de Silva et al., 2011; Perea, 2018) for these 1-cocycles (Figure 5B). These coordinates describe the topological space of the torus and should map back onto the rhombic unit cell that tiles physical space. To explore this relationship, we project the entire time series of neural activities onto these coordinates. For each neuron, we find the data points for which that neuron is the most active within the population. These points are clustered and define firing fields in topological space (Figure 5C). The center of masses of these clusters are used to evaluate distances between grid cells; these topological distances are highly correlated with the physical distances between grid offsets within the rhombic unit cell (Figure 5D). Thus, the topological coordinates defined in neural activity space indeed capture the organization of grid cells in physical space.

Furthermore, persistent cohomology can leverage the mapping between physical and topological spaces to decode trajectories in the former by trajectories in the latter (Figure 5E). To do so, we trace the circular coordinates depicted in Figure 5C through time to form a raw trajectory through topological space. We then unshear it by 60° and unfold this trajectory by identifying large jumps with wrapping through the boundary to produce a reconstructed segment. These steps do not require knowledge of the animal's true trajectory (although some general expectations about the animal's motion are required for unshearing; see Methods section 4.5 for details). We find that this reconstruction can be translated, rotated, reflected, and/or uniformly scaled to match the true trajectory very well. Without spiking noise, almost all reconstructions deviate from the true trajectory by <4 cm averaged across time, which is much less than the enclosure's diameter of 180 cm (Figures 5F,G). This error decreases with more neurons or more timepoints in the simulated recording. Similar trends are observed with the introduction of Poisson noise that mimics spiking noise, but more neurons are required, more outliers with poor fit are observed, and geometric subsampling cannot be used to improve computational tractability (Figures 5H,I).

### 2.4. Persistent Cohomology for Mixtures of Neural Populations

**Figure 6:**
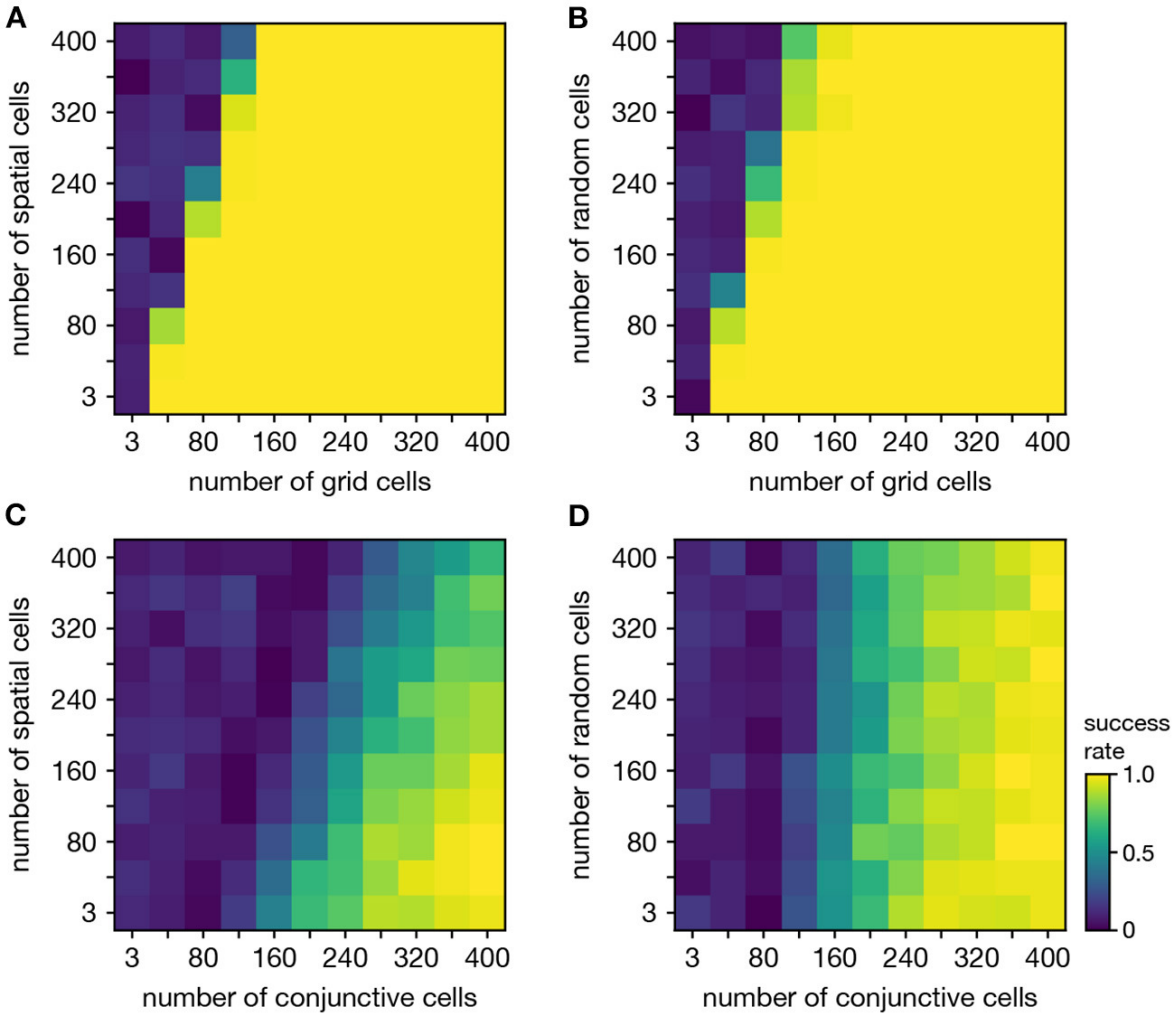
Persistent cohomology in combinations of periodic and non-periodic neural populations. Success is defined by observing the number of persistent 1-cocycles expected from the periodic population, which is two for grid cells and three for conjunctive cells. **(A)** Grid cells from module 1 and non-grid spatial cells. **(B)** Grid cells from module 1 and random cells. **(C)** Conjunctive cells from module 1 and non-grid spatial cells. **(D)** Conjunctive cells from module 1 and random cells.

Persistent cohomology can discover topological structure in mixtures of neural populations. When neurons are recorded from a periodic neural population and a non-periodic neural population, the latter adds additional dimensions to the point cloud embedding, but the topological structure contained within the former may persist. We test if persistent cohomology can recover this information in mixed datasets with neurons from both a periodic population (either grid or conjunctive) and a non-periodic population (either non-grid spatial or random). Reliable discovery of the torus formed by grid cells is possible when the number of spatial or random cells is less than twice the number of grid cells (Figures 6A,B). Detection of the 3-torus formed by conjunctive cells requires more neurons, but it can also be reliably achieved in the presence of non-periodic populations (Figures 6C,D). Thus, persistent cohomology demonstrates robustness to the inclusion of non-periodic populations. The size of the non-periodic population that can be tolerated increases with the size of the periodic population.

**Figure 7:**
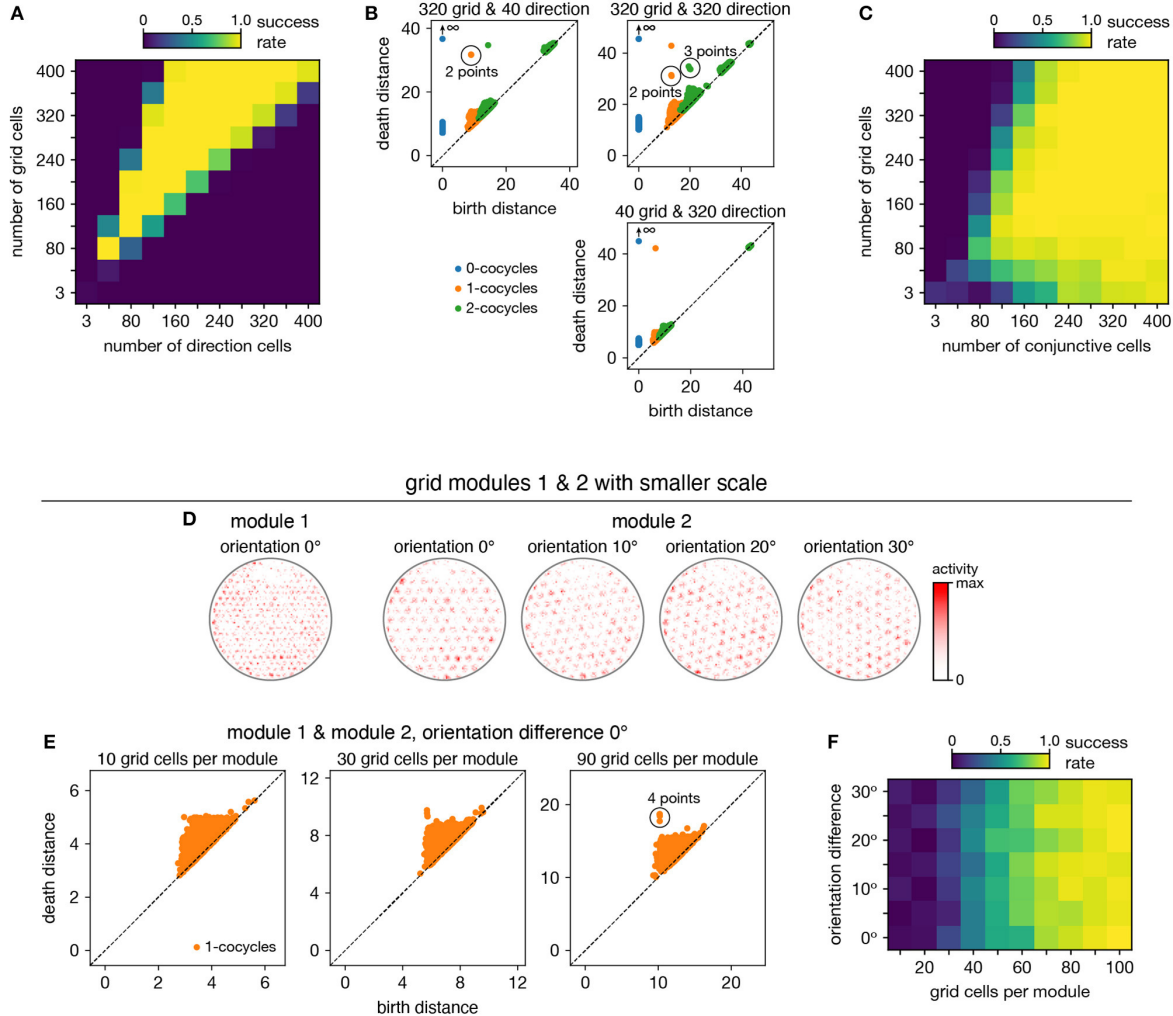
Persistent cohomology in combinations of periodic neural populations. Success is defined by observing the number of persistent 1-cocycles expected from the product topology. **(A,B)** Discovering the 3-torus for grid cells from module 1 and head direction cells. **(A)** Success rates. **(B)** Example persistence diagrams. If one population contributes many more neurons than the other, persistent cohomology detects the topology of the predominant population. **(C)** Success rates for discovering the 3-torus for grid cells and conjunctive cells, both from the same module 1. **(D–F)** Combining grid cells from two modules with smaller scales. **(D)** Positional activity maps for example neurons from module 1 and four different orientations of module 2. **(E)** Example persistence diagrams. **(F)** Success rates for discovering the 4-torus.

When neurons are recorded from multiple periodic neural populations, their structures are preserved within projected subspaces of high-dimensional activity space. We explore persistent cohomology in this scenario by forming mixed datasets with neurons from two periodic populations. When the two populations respond to unrelated signals, such as grid and head direction cells—the combined topological space should be the Cartesian product of those of the separate populations. Indeed, that persistent cohomology can discover the resultant 3-torus at intermediate mixing ratios (Figures 7A,B). If one population contributes many more neurons—and thus embedding dimensions—than the other, we instead detect the corresponding single-population structure (Figure 7B).

When the two populations respond to related signals, such as grid and conjunctive cells—the activity space of one is contained in the activity space of the other. Grid cells and conjunctive cells from the same module encode position with the same toroidal structure; they both tile space with the same rhombic unit cell of neural activity. In addition, the conjunctive population encodes direction with a circular topology. Thus, the mixed dataset should span a 3-torus, which can be detected by persistent cohomology (Figure 7C). For reliable discovery, at least ≈120 conjunctive cells and at least ≈240 total neurons are required. However, discovery of the product topology is disrupted if the number of grid cells exceeds the number of conjunctive cells by more than a factor of ≈1.5. Thus, persistent cohomology can best detect product topologies when the mixed dataset is not dominated by one population.

Finally, we consider the case of mixing grid cells from multiple modules. Grid modules have different rhombic unit cells with different scales and orientations, so they map the same physical space onto different topological coordinates. Thus, a mixed dataset from two different modules should exhibit the product topology of two 2-tori, which is the 4-torus. However, we are unable to reliably discover this structure using the grid modules illustrated in Figure 1A; they are too sparse. To produce a point cloud that embeds the toroidal structure for one grid module, the animal trajectory should densely sample its rhombic unit cell. This is achieved since the enclosure contains many unit cells. However, to produce a point cloud that embeds the 4-torus formed by two grid modules, the animal trajectory should densely sample all combinations of unit cells. This is not achieved by the grid modules illustrated in Figure 1A because the enclosure contains too few rhombic unit cells for them to overlap in many different configurations.

Thus, we generate two grid modules separated by the same scale ratio as in Figure 1A, but with one-fourth of its scale (Figure 7D). In addition, we explore different relative orientations between the modules by generating different orientations for module 2. Notably, these scale ratios and orientation differences are not chosen such that the two rhombic unit cells would share a simple geometric relationship with each other (Kang and Balasubramanian, 2019), which would limit their possible overlap configurations and collapse the expected 4-torus structure to a 2-torus. As we include more neurons from both modules into our dataset, we see that four persistent 1-cocycles eventually emerge from the points close to the diagonal that represent sampling noise (Figure 7E). The success of persistent cohomology is independent of the orientation difference between the two modules (Figure 7F). Note that if we obtained independent activity samples from each module, combined datasets formed from the original grid modules with larger scales should exhibit 4-torus structure. However, samples taken from a single animal trajectory are not independent across modules, so smaller grid scales (or equivalently, larger environments) are required to fully sample the 4-torus structure.

## 3. Discussion

We demonstrate that persistent cohomology can discover topological structure in simulated neural recordings with as few as tens of neurons from a periodic neural population (Figure 3). From this structure, it can decode the trajectory of the animal using only the time series of neural activities (Figure 5). It can also discover more complex topological structures formed by combinations of periodic neural populations if each population is well-represented within the dataset (Figure 7).

By comprehensively adjusting a wide range of parameters related to grid cells, we find that persistent cohomology generally behaves in three different ways with respect to parameter variation. First, topological discovery can be unaffected by some manipulations, such as combining grid cells into multi-neuron units and changing global geometric features, such as enclosure geometry and lattice aspect ratio (Figure 4). Second, topological discovery may be impeded in a way that is counteracted by increasing neuron number. Spiking noise, small field sizes, and inclusion of non-periodic populations are examples of parameters that exhibit this behavior (Figures 4, 6). Third, topological discovery can fail catastrophically in certain parameter regimes without the possibility of recovery by including more neurons. This happens if tuning curves are inherently too variable or if discovery of product topologies are desired when one neural population vastly outnumbers the other (Figures 4, 7). These conclusions can help researchers understand and overcome obstacles to topological discovery with persistent (co)homology and may guide its use across a variety of neural systems.

We have characterized the capabilities of persistent cohomology using simple simulated data, but our results may generalize to real neural data. A key requirement for generalization is the separation of two timescales. The macroscopic timescale at which topological structures are explored—here, the time required to traverse a rhombic unit cell of a grid module or 360° of head direction—must be much longer than the microscopic timescale at which neuronal activity is generated. This enables us to coarse-grain over spikes and describe the activity by a firing rate. Indeed, the inputs to our analysis pipeline are firing rates over 0.2 s time bins, which averages over many neurophysiological processes, including major neural oscillations found in the hippocampal region (Lisman and Jensen, 2013). Similar forms of coarse-graining were used by Rybakken et al. (2019), Chaudhuri et al. (2019), and Gardner et al. (2021) to successfully apply persistent (co)homology to experimental recordings in the spatial representation system.

For comparison, we attempt to modify manifold learning algorithms to enable discovery and interpretation of topological structure (Supplementary Material and Supplementary Figure 1). We find that Isomap (Tenenbaum et al., 2000) followed by Independent Components Analysis (ICA) (Hyvärinen, 1999) can successfully identify and decode from toroidal structure with modified grid cells whose tuning curves exhibit a square lattice. Unlike persistent cohomology, it does not work for triangular lattices and does not consider topological features with dimensionality >1. UMAP (McInnes et al., 2018) can be used to embed grid cell data directly into a 2-dimensional space with a toroidal metric, but we find that its coordinates do not correspond well to the physical grid periodicity. In short, persistent cohomology performs better and requires adjusting fewer parameters for topological data analysis compared to these alternative methods, which were not designed for such analysis. Note that Chaudhuri et al. (2019) formulate an alternative method for decoding topologically encoded information; they also use persistent homology for discovery of topological structure.

The application of persistent (co)homology to neuroscience data is still in its developing stages. In addition to the research on spatial representation circuits described above (Chaudhuri et al., 2019; Rybakken et al., 2019; Gardner et al., 2021), notable lines of work include: simulations of hippocampal place cells in spatial environments with non-trivial topology (Dabaghian et al., 2012; Spreemann et al., 2015; Babichev et al., 2018, 2019; Chowdhury et al., 2018); analysis of EEG signals, for classification and detection of epileptic seizures (Piangerelli et al., 2018; Wang et al., 2018) and for construction of functional networks in a mouse model of depression (Khalid et al., 2014); inferring intrinsic geometric structure in neural activity (Giusti et al., 2015); and detection of coordinated behavior between human agents (Zhang et al., 2020). There is potential for persistent (co)homology to provide insight to a wide range of neural systems. Topological structures generally can be found wherever periodicities exist. These periodicities can take many forms, such as the spatial periodicities in our work, temporal regularities in neural oscillations, motor patterns, and neural responses to periodic stimuli.

The toolbox of topological data analysis has more methods beneficial to the analysis of neural data. The methods described in this paper, including geometric subsampling, are sensitive to outliers. This problem can be addressed within the same framework of persistent cohomology by using the distance-to-a-measure function (Chazal et al., 2011). In practice, this would translate into a slightly more elaborate construction (Guibas et al., 2013) of the Vietoris–Rips complex. Furthermore, our analysis pipeline benefits from having neural activity embedded in a high dimensional space, i.e., from having many more neurons than the intrinsic dimensions of the recovered tori. It is possible to adapt this technique to the regime of limited neural recordings (even to a single neuron) by using time-delay embeddings (Takens, 1981). However, for spatial populations, such a technique would require control over the smoothness of the animal's trajectory, which may not be feasible in practice. Also, the method we present cannot make inferences on network topology. If connectivity information were present in neural activities, they should appear on fast timescales related to synaptic integration, action potential propagation, and synaptic delay. By averaging neural activity into 0.2 s time bins, we destroy this information, but it is possible that a modified method may access it.

Our results also suggest research directions in topological data analysis. Throughout the paper, we relied on 1-dimensional persistent cohomology to infer whether we recovered a particular torus. But that is a relatively weak method: many topological spaces have cohomology groups of the same dimension. Although the trajectories that we recover via circular coordinates serve as a convincing evidence that we are indeed recovering the tori, it is possible to confirm this further by exploiting cup product structure in cohomology, which is a particular kind of a topological operation that turns cohomology into a ring. Computing a “persistent cup product” would provide additional evidence about the structure of the recovered spaces.

## 4. Methods

### 4.1. Generating Neural Recordings

#### 4.1.1. Animal Trajectory

We simulate the simultaneous recording of neurons from a rat as it explores a circular enclosure of diameter 1.8 m. We use 1,000 s from a trajectory extracted from an experimental animal (Hafting et al., 2005; Burak and Fiete, 2009). This trajectory is provided as velocities sampled at 0.5 ms intervals along with the initial position. We average these to positions and directions at 0.2 s intervals as follows: the position of the animal is simply the average position within each 0.2 s time bin, and the direction of the animal is the circular mean of the velocity vector angle within each 0.2 s time bin. We ignore the distinction between body direction and head direction.

#### 4.1.2. Periodic Neural Populations

We generate tuning curves as a function of position and/or direction for each neuron. These localized tuning curves are based on a shifted and truncated cosine function:

(1) $$\begin{array}{l} f\left(z\right)=\{\begin{array}{cc} \frac{1+\cos\pi \;z}{2} & |z|<1\\ 0 & |z|\geq 1. \end{array} \end{array}$$

For each grid module, we set a scale *l* and an orientation ϕ. This defines a transformation matrix from the space of phases $\left[-\frac{1}{2},\frac{1}{2}\right)\times \left[-\frac{1}{2},\frac{1}{2}\right)$ to the rhombic unit cell of the grid module in physical space:

(2) $$\begin{array}{l} \text{A}=l\left(\begin{array}{cc} \cos\phi & \cos\left(\phi +\frac{\pi }{3}\right)\\ \sin\phi & \sin\left(\phi +\frac{\pi }{3}\right) \end{array}\right). \end{array}$$

Unless otherwise specified, we use *l* = 40 cm and ϕ = 0. The inverse of this matrix A^−1^ maps the rhombic unit cell onto the space of phases. We also define ||·|| as the vector norm and 〈*a*〉_*m*_ ≡ (*a* + *m* mod 2*m*) − *m* as the shifted modulo operation. The tuning curve of a grid cell as a function of position x is then

(3) $$\begin{array}{l} s_{\text{grid}}\left(\text{x};\text{b}\right)=f\left(\frac{1}{0.45l}\left||\text{A}{\langle {\text{A}}^{-1}\text{x}-\text{b}\rangle }_{1/2}\right||\right) \end{array}$$

Each grid cell is shifted by a uniformly random phase offset b. The full width at half maximum of each grid field is 0.45*l*. In physical space, the offset of a grid cell is Ab, where integers can be added to either component of b; the shortest distance between these offsets for two grid cells is the physical distance shown in Figure 5D.

The tuning curve of a head direction cell as a function of direction θ is

(4) $$\begin{array}{l} s_{\text{dir}}\left(\theta ;c\right)=f\left(4{\langle \theta -c\rangle }_{\pi }\right). \end{array}$$

Each head direction cell is shifted by a uniformly random direction offset *c*. The full width at half maximum of the head direction field is π/2.

The tuning curve of a conjunctive cell is simply the product

(5) $$\begin{array}{l} s_{\text{conj}}\left(\text{x},\theta ;\text{b},c\right)=s_{\text{grid}}\left(\text{x};\text{b}\right)s_{\text{dir}}\left(\theta ;c\right). \end{array}$$

Each conjunctive cell has uniformly random offsets b and *c*.

#### 4.1.3. Non-periodic Neural Populations

For non-grid spatial cells, we generate tuning curves as a function of position for each neuron of the form

(6) $$\begin{array}{l} s_{\text{spatial}}\left(\text{x}\right)=\frac{1}{2}\left({\text{e}}^{-\|\text{x}-{\text{d}}_{1}{\|}^{2}/2\sigma^{2}}+{\text{e}}^{-\|\text{x}-{\text{d}}_{2}{\|}^{2}/2\sigma^{2}}\right), \end{array}$$

where σ = 40 cm and the d_*i*_'s are chosen uniformly randomly between (0, 0 cm) and (180, 180 cm).

For random neurons, we obtain activity time series by sampling from a distribution every 2 s, or 10 timepoints, and interpolating in between using cubic polynomials. The distribution is Gaussian with mean 0 and width 0.5, truncated between 0 and 1.

#### 4.1.4. From Tuning Curves to Time Series

To obtain activity time series for all populations except for random neurons, we apply the tuning curves to each trajectory timepoint. Whenever the velocity decreases below 5 cm/s, we set the activity to be 0. This threshold simulates the behavior of neurons in the hippocampal region that exhibit high activity during locomotion and low activity during idle periods (Sargolini et al., 2006; Kropff et al., 2015; Hinman et al., 2016).

#### 4.1.5. Multi-Neuron Units for Grid Cells (Figures 4A,B)

We generate multi-neuron units (Figure 4) by linearly combining activity time series from multiple grid cells. Each mixing coefficient is chosen from a uniform random distribution between 0 and 1. The activity is then normalized by the sum of squares of the mixing coefficients.

#### 4.1.6. Spiking Noise for Grid Cells (Figures 4C,D)

The activities described above are dimensionless, and we typically do not need to assign a scale because we divide each time series by its mean before applying persistent cohomology. To create spiking noise, however, we must set the firing rate. We linearly rescale the rate given by Equation (3):

(7) $$\begin{array}{l} \lambda =0.4+7.6s_{\text{grid}}. \end{array}$$

This sets the maximum firing rate to be 8 and creates a baseline rate of 0.4; with 0.2 s time bins, these values correspond to 40 and 2 Hz, respectively. However, we still set the firing rate to 0 Hz when the animal's velocity decreases below 5 cm/s.

Using λ, we generate Poisson-like spiking noise with different levels of variability (Figure 4). At each timestep, the noisy activity is given by

(8) $$\begin{array}{l} s_{\text{noisy}}=F\cdot X,\;X\sim \text{Pois}\left(\lambda /F\right). \end{array}$$

*F* sets the Fano factor of the random process, which is its variance divided by its mean (for any given λ). The *F* = 1 case corresponds to a Poisson random process; *F* < 1 implies sub-Poissonian noise and *F* > 1 implies super-Poissonian noise.

#### 4.1.7. Generating Grid Cells With Various Properties (Figures 4E–K)

- Square enclosure (Figure 4E): We use 1,000 s from a trajectory extracted from an experimental animal in a square enclosure of width 1.5 m (Stensola et al., 2012), binned in the same way as for the circular enclosure (section 4.1.1). To change the lattice orientation, we use a non-zero value for ϕ in Equation (2).
- Scale factor (Figure 4F): Grid scale is modified by changing *l* in Equation (2). The scale factor is *l* divided by the diameter of the enclosure, 1.8 m.
- Field size factor (Figure 4G): Field size is modified by replacing 0.45 in Equation (3) by the field size factor. In other words, the field size factor multiplied by the grid scale is the full width at half maximum of each grid field.
- Aspect ratio (Figure 4H): The grid lattice is elongated to an aspect ratio ε ≥ 1, which is the ratio between the major and minor axes of the ellipse that circumscribes each hexagonal lattice domain. This is accomplished by replacing the transformation matrix in Equation (2) with
  (9) $$\begin{array}{l} \text{A}=l\cdot \frac{\sqrt{3}}{2}\sec2\psi \left(\begin{array}{cc} 2\cos\psi \cos\phi & \cos\left(\phi +\psi \right)\\ 2\cos\psi \sin\phi & \sin\left(\phi +\psi \right) \end{array}\right), \end{array}$$
  where ψ ≤ 60° is the angle between the two lattice vectors. ψ is related to ε by
  (10) $$\begin{array}{l} \psi =\mathrm{arcsec}\left(\frac{3}{\varepsilon }-1\right). \end{array}$$
  When $\varepsilon =3/\left(1+\sqrt{2}\right)\approx 1.24$, ψ = 45° and the lattice becomes square.
- Field jitter (Figure 4I): Jitter in the position of grid fields is introduced by shifting each field in physical space by
  (11) $$\begin{array}{l} U_{1}l\left(\begin{array}{c} \cos\phi \\ \sin\phi \end{array}\right)+U_{2}l\left(\begin{array}{c} \cos\left(\phi +\frac{\pi }{3}\right)\\ \sin\left(\phi +\frac{\pi }{3}\right) \end{array}\right), \end{array}$$
  where *l* is the grid scale, ϕ is the grid orientation, and *U*_*i*_ ~ Unif(−field jitter, field jitter).
- Field variability (Figure 4J): Variability across grid fields is introduced by choosing random numbers *U*_*ij*_ ~ Unif(−field var., field var.) at 20 cm-intervals throughout the enclosure for each grid cell. A linear interpolation is constructed for these points, which is smoothed by a Gaussian filter of width 20 cm. This resulting random function is added to the standard tuning curve in Equation (3), and all values under 0 are clipped to 0.
- Lattice variability (Figure 4K): Variability in lattices across grid cells is introduced by randomly perturbing the transformation matrix for each grid cell (Equation 2) according to
  (12) $$\begin{array}{l} \text{A}=\left(\begin{array}{cc} 1+W_{11} & W_{12}\\ W_{21} & 1+W_{22} \end{array}\right)l\left(\begin{array}{cc} \cos\phi & \cos\left(\phi +\frac{\pi }{3}\right)\\ \sin\phi & \sin\left(\phi +\frac{\pi }{3}\right) \end{array}\right), \end{array}$$
  where
  (13) $$\begin{array}{l} W_{ij}\sim \text{Unif}\left(-\text{lattice var.},\text{lattice var.}\right). \end{array}$$

### 4.2. Neural Activity Maps

We construct activity maps for each neuron as a function of position or direction. To do so, we simply tally the total amount of activity in each positional or directional bin. Note that these maps do not depict firing rate because we do not divide by the occupancy of each bin; we decided against this in order to show the activity experienced through the animal trajectory.

### 4.3. Processing Neural Recordings

For each neuron, we first divide its activity at every timepoint by its mean activity. We then delete all timepoints whose neural activities are all smaller than a small limit 1 × 10^−4^. This simple procedure removes points at the origin that we do not expect to participate in topological structures.

To improve computational efficiency, we reduce the number of input points while preserving their topological structure by applying a geometric subsampling strategy. We pick the first point at random, and then iteratively add a point to our subsample that is the furthest away from the already chosen points. Specifically, if *P* is the input point set and *Q*_*i*_ is the subsample after *i* iterations, we form *Q*_*i*+1_ by adding the point *q*_*i*+1_ chosen as

(14) $$\begin{array}{l} \arg\underset{p\in P}{\max}\underset{q\in Q_{i}}{\min}\|p-q\|. \end{array}$$

Figure 1B illustrates a result of this strategy. By construction, the subsample *Q*_*i*_ forms an ε_*i*+1_-net of the input point sample, which means the largest distance from any input point to the nearest point of the subsample does not exceed $\varepsilon_{i+1}=\underset{q\in Q_{i}}{\min}\|q_{i+1}-q\|$. Because persistent cohomology is stable, this guarantees that the persistence diagram we compute for the subsample *Q*_*i*_ is at most ε_*i*+1_ away, in bottleneck distance (Cohen-Steiner et al., 2007), from the persistence diagram of the full point set *P*. We generally select 1,000 points through this process. The results in Figures 5H,I were obtained by applying persistent cohomology on the full dataset without subsampling.

To measure success rate, we apply persistent cohomology on 100 replicate datasets and measure the proportion of successes as determined by the largest-gap heuristic.

### 4.4. Applying Persistent Cohomology

We refer the reader to extensive literature on persistent (co)homology (Edelsbrunner and Harer, 2010; Edelsbrunner and Morozov, 2017) for the full details. More details on the involved constructions are presented in the Supplementary Material. Here, we only briefly mention some of them. For technical reasons—both to recover the circular coordinates and for computational speed—we work with persistent cohomology, which is dual to persistent homology, which a reader might be more familiar with.

To recover the topology of the space sampled by a point set *P*, we construct a Vietoris–Rips simplicial complex. Given a parameter *r*, Vietoris–Rips complex consists of all subsets of the point set *P*, in which every pair of points is at most *r* away from each other,

(15) $$\begin{array}{l} \mathrm{VR}\left(P,r\right)=\left\{\sigma \subseteq P|\|p-q\|\leq r\forall p,q\in \sigma \right\}. \end{array}$$

The cohomology group, H^*k*^(VR(*P, r*)), defined formally in the Supplementary Material, is an algebraic invariant that describes the topology of the Vietoris–Rips complex. Its rank, called the *k*-th Betti number, counts the number of “holes” in the complex.

As we vary the radius *r* in the definition of the Vietoris–Rips complex, the simplicial complexes nest: VR(*P, r*_1_) ⊆ VR(*P, r*_2_), for *r*_1_ ≤ *r*_2_. The restriction of the larger complex to the smaller induces a linear map on cohomology groups, and all such maps form a sequence:

(16) $$\begin{array}{l} {\text{H}}^{k}\left(\mathrm{VR}\left(P,r_{1}\right)\right)\leftarrow {\text{H}}^{k}(\mathrm{VR}\left(P,r_{2}\right)\leftarrow {\text{H}}^{k}\left(\mathrm{VR}\left(P,r_{3}\right)\right)\leftarrow \ldots \end{array}$$

It is possible to track when cohomology classes (i.e., “holes”) appear and disappear in this sequence. Recording all such birth–death pairs (*r*_*i*_, *r*_*j*_), we get a persistence diagram, which completely describes the changes in the sequence of cohomology groups.

For a persistent class, i.e., one with a large difference between birth and death, de Silva et al. (2011) describe a procedure for turning it into a map from the input data points into a circle, which assigns a circular coordinate to each data point. Perea (2018) extends that procedure to allow computation of persistent cohomology on a subsample of the data, e.g., the geometric subsample mentioned in the previous subsection.

### 4.5. Reconstructing Animal Trajectory From Circular Coordinates

The process for obtaining circular coordinates outlined in the previous subsection (and presented in greater detail in the Supplementary Material) outputs one value between 0 and 2π for each persistent 1-cocycle at each timepoint. A grid module yields two persistent 1-cocycles, so our circular coordinates form a vector u_*t*_ = (*u*_*t*1_, *u*_*t*2_) at each timepoint *t* = 1, …, *T*. We divide each coordinate value by 2π so that each *u*_*ti*_ ∈ [0, 1). No matter the recording duration used to obtain circular coordinates, we only reconstruct the first 100 s of the animal trajectory.

We first perform a preliminary unfolding of the circular coordinates. We calculate all the difference vectors between adjacent timepoints and cancel out changes by more than ±1/2:

(17) $$\begin{array}{l} \Delta u_{ti}^{\text{unfolded}}={\langle u_{ti}-u_{t-1,i}\rangle }_{1/2},\\ \text{ where }{\langle a\rangle }_{1/2}\equiv \left(a+1/2\text{ mod }1\right)-1/2. \end{array}$$

Next, we seek to unshear the coordinates. The rhombic unit cell in physical space is sheared by 30° relative to the orthogonal axes of topological space. We wish to apply this transformation to the difference vectors to restore the unsheared trajectory. There are two possible unshearing matrices

(18) $$\begin{array}{l} {\text{S}}^{\pm }=\left(\begin{array}{cc} \cos0 & \cos\left(\frac{\pi }{2}\pm \frac{\pi }{6}\right)\\ \sin0 & \sin\left(\frac{\pi }{2}\pm \frac{\pi }{6}\right), \end{array}\right) \end{array}$$

and we could perform the rest of the analysis for both transformations separately, knowing that one trajectory is unsheared and the other is doubly shared. Instead, we assume knowledge that the animal is exploring an open field environment in which all directions of motion should generally be sampled uniformly. We calculate the covariance matrix for both sets of transformed difference vectors:

(19) $$\begin{array}{l} \Sigma_{ij}^{\pm }=\frac{1}{T}\underset{t}{\sum }\Delta u_{ti}^{\pm }\Delta u_{tj}^{\pm },\text{ where }\Delta {\text{u}}_{t}^{\pm }={\text{S}}^{\pm }\Delta {\text{u}}_{t}^{\text{unfolded}}. \end{array}$$

The proper unshearing S produces the covariance matrix whose ratio of eigenvalues is closest to 1. This heuristic could be changed assuming different statistics of animal motion, for example those corresponding to a linear track.

After identifying the unshearing matrix S, we return to the raw coordinates and apply this transformation first, before unfolding:

(20) $$\begin{array}{l} \Delta {\text{u}}_{t}^{\text{unsheared}}=\text{S}\left({\text{u}}_{t}-{\text{u}}_{t-1}\right). \end{array}$$

Since shear transformations change distances, we reevaluate our unfolding. We compare the norm of every difference vector $\Delta {\text{u}}_{t}^{\text{unsheared}}$ to its norm after possible unfoldings along the unsheared lattice vectors given by the columns of S. The shortest vector at each timepoint is the reconstructed difference $\Delta {\text{u}}_{t}^{\text{recon}}$, and the reconstructed trajectory segment shown in Figure 5E is their accumulation ${\text{u}}_{t}^{\text{recon}}=\underset{t}{\sum }\Delta {\text{u}}_{t}^{\text{recon}}$.

So far, this reconstruction has not incorporated detailed information about the animal trajectory. To judge its quality, we now fit the reconstruction to the true segment of animal trajectory x_*t*_ through rigid transformation and uniform scaling. We first determine whether or not the reconstruction needs to be unreflected. To judge this, we calculate the signed vector angles between adjacent steps for both the reconstruction and the true trajectory

(21) $$\begin{array}{l} \Delta \theta_{t}^{\text{recon}}={\langle \arctan\left(\Delta u_{t2}^{\text{recon}}/\Delta u_{t1}^{\text{recon}}\right)-\arctan\left(\Delta u_{t-1,2}^{\text{recon}}/\Delta u_{t-1,1}^{\text{recon}}\right)\rangle }_{\pi }\\ \;\Delta \theta_{t}={\langle \arctan\left(\Delta x_{t2}/\Delta x_{t1}\right)-\arctan\left(\Delta x_{t-1,2}/\Delta x_{t-1,1}\right)\rangle }_{\pi }, \end{array}$$

where 〈*a*〉_π_ ≡ (*a* + π mod 2π) − π. If the mean square difference between $-\Delta \theta_{t}^{\text{recon}}$ and Δθ_*t*_ is less than that between $\Delta \theta_{t}^{\text{recon}}$ and Δθ_*t*_, then we reflect our reconstruction ${\text{u}}_{t}^{\text{oriented}}=\left(\begin{array}{cc} 1 & 0\\ 0 & -1 \end{array}\right){\text{u}}_{t}^{\text{recon}}$. Otherwise, ${\text{u}}_{t}^{\text{oriented}}={\text{u}}_{t}^{\text{recon}}$.

Finally, we fit the reconstruction to the true trajectory segment by minimizing the mean squared error after uniform scaling *a*, translation b and rotation θ:

(22) $$\begin{array}{l} \underset{a,\text{b},\theta }{\min}\underset{t}{\sum }{\left\|{\text{x}}_{t}-a\left(\begin{array}{cc} \cos\theta & -\sin\theta \\ \sin\theta & \cos\theta \end{array}\right){\text{u}}_{t}^{\text{oriented}}+\text{b}\right\|}^{2}. \end{array}$$

Note that we chose to try particular unshearing matrices in Equation (18) and use the general assumption of isotropic animal motion to select between them, all before fitting the reconstruction to the actual animal trajectory in Equation (22). These choices were designed to obtain the most faithful reconstruction without using the animal trajectory, which is the information that we would like to infer. This process assumes knowledge of the angle between directions of periodicity, which is ~60° for grid modules. Moreover, it also relies on the circular coordinates implementation (explained in greater detail in Supplementary Material) selecting two of the shortest non-parallel vectors between lattice vertices as its basis—that selection is not guaranteed, but empirically, it frequently occurs in our case, as indicated by low reconstruction errors in Figure 5. An alternative method for unshearing before comparison with the true trajectory is to find a transformation matrix that yields a covariance matrix for the transformed difference vectors (analogously to Equation 19) with a desired ratio of eigenvalues. Otherwise, if a sheared reconstruction is sufficient, unshearing can be skipped.

## Data Availability Statement

The datasets and source code presented in this study can be found in online repositories. The names of the repository/repositories and accession number(s) can be found at: https://louiskang.group/repo.

## Author Contributions

LK and DM conceptualized the study and wrote the paper. LK, BX, and DM developed the methods, calculated the persistent cohomology, and analyzed the results. LK generated the neural data. All authors contributed to the article and approved the submitted version.

## Conflict of Interest

The authors declare that the research was conducted in the absence of any commercial or financial relationships that could be construed as a potential conflict of interest.

## References

[B1] Babichev A. Morozov D. Dabaghian Y. (2018). Robust spatial memory maps encoded by networks with transient connections. PLoS Comput. Biol. 14:e1006433. 10.1371/journal.pcbi.1006433 30226836 PMC6161922

[B2] Babichev A. Morozov D. Dabaghian Y. (2019). Replays of spatial memories suppress topological fluctuations in cognitive map. Netw. Neurosci. 3, 707–724. 10.1162/netn_a_00076 31410375 PMC6663216

[B3] Burak Y. Fiete I. R. (2009). Accurate path integration in continuous attractor network models of grid cells. PLoS Comput. Biol. 5:e1000291. 10.1371/journal.pcbi.1000291 19229307 PMC2632741

[B4] Chaudhuri R. Gerçek B. Pandey B. Peyrache A. Fiete I. (2019). The intrinsic attractor manifold and population dynamics of a canonical cognitive circuit across waking and sleep. Nat. Neurosci. 22, 1512–1520. 10.1038/s41593-019-0460-x 31406365

[B5] Chazal F. Cohen-Steiner D. Mérigot Q. (2011). Geometric inference for measures based on distance functions. Foundat. Comput. Math. 11, 733–751. 10.1007/s10208-011-9098-0

[B6] Chowdhury S. Dai B. Mémoli F. (2018). The importance of forgetting: limiting memory improves recovery of topological characteristics from neural data. PLoS ONE 13:e0202561. 10.1371/journal.pone.0202561 30180172 PMC6122934

[B7] Cohen-Steiner D. Edelsbrunner H. Harer J. (2007). Stability of persistence diagrams. Discrete Comput. Geometry 37, 103–120. 10.1007/s00454-006-1276-5

[B8] Curto C. (2016). What can topology tell us about the neural code? Bull. Am. Math. Soc. 54, 63–78. 10.1090/bull/1554

[B9] Dabaghian Y. Mémoli F. Frank L. Carlsson G. (2012). A topological paradigm for hippocampal spatial map formation using persistent homology. PLoS Comput. Biol. 8:e1002581. 10.1371/journal.pcbi.1002581 22912564 PMC3415417

[B10] de Silva V. Morozov D. Vejdemo-Johansson M. (2011). Persistent cohomology and circular coordinates. Discrete Comput. Geometry 45, 737–759. 10.1007/s00454-011-9344-x

[B11] Diehl G. W. Hon O. J. Leutgeb S. Leutgeb J. K. (2017). Grid and nongrid cells in medial entorhinal cortex represent spatial location and environmental features with complementary coding schemes. Neuron 94, 83–92.e6. 10.1016/j.neuron.2017.03.004 28343867 PMC5444540

[B12] Edelsbrunner H. Harer J. (2010). Computational Topology: An Introduction. Providence, RI: American Mathematical Society. 10.1090/mbk/069

[B13] Edelsbrunner H. Morozov D. (2017). “Persistent homology,” in Handbook of Discrete and Computational Geometry, eds J. E. Goodman, J. O'Rourke, and C. D. Tóth (Boca Raton, FL: CRC Press), 637–661.

[B14] Fiete I. R. Burak Y. Brookings T. (2008). What grid cells convey about rat location. J. Neurosci. 28, 6858–6871. 10.1523/JNEUROSCI.5684-07.2008 18596161 PMC6670990

[B15] Gallego J. A. Perich M. G. Miller L. E. Solla S. A. (2017). Neural manifolds for the control of movement. Neuron 94, 978–984. 10.1016/j.neuron.2017.05.025 28595054 PMC6122849

[B16] Gao P. Ganguli S. (2015). On simplicity and complexity in the brave new world of large-scale neuroscience. Curr. Opin. Neurobiol. 32, 148–155. 10.1016/j.conb.2015.04.003 25932978

[B17] Gardner R. J. Hermansen E. Pachitariu M. Burak Y. Baas N. A. Dunn B. A.. (2021). Toroidal topology of population activity in grid cells. bioRxiv [Preprint]. 10.1101/2021.02.25.432776 PMC8810387 35022611

[B18] Giusti C. Pastalkova E. Curto C. Itskov V. (2015). Clique topology reveals intrinsic geometric structure in neural correlations. Proc. Natl. Acad. Sci. U.S.A. 112, 13455–13460. 10.1073/pnas.1506407112 26487684 PMC4640785

[B19] Guibas L. Morozov D. Mérigot Q. (2013). Witnessed k-distance. Discrete Comput. Geometry 49, 22–45. 10.1007/s00454-012-9465-x

[B20] Hafting T. Fyhn M. Molden S. Moser M. B. Moser E. I. (2005). Microstructure of a spatial map in the entorhinal cortex. Nature 436, 801–806. 10.1038/nature03721 15965463

[B21] Hardcastle K. Maheswaranathan N. Ganguli S. Giocomo L. M. (2017). A multiplexed, heterogeneous, and adaptive code for navigation in medial entorhinal cortex. Neuron 94, 375–387.e7. 10.1016/j.neuron.2017.03.025 28392071 PMC5498174

[B22] Hinman J. R. Brandon M. P. Climer J. R. Chapman G. W. Hasselmo M. E. (2016). Multiple running speed signals in medial entorhinal cortex. Neuron 91, 666–679. 10.1016/j.neuron.2016.06.027 27427460 PMC4976037

[B23] Hyvärinen A. (1999). Fast and robust fixed-point algorithms for independent component analysis. IEEE Trans. Neural Netw. 10, 626–634. 10.1109/72.761722 18252563

[B24] Jazayeri M. Afraz A. (2017). Navigating the neural space in search of the neural code. Neuron 93, 1003–1014. 10.1016/j.neuron.2017.02.019 28279349

[B25] Johnson W. B. Lindenstrauss J. (1984). Extensions of lipschitz mappings into a hilbert space. Contemp. Math. 26:1. 10.1090/conm/026/737400

[B26] Jun J. J. Steinmetz N. A. Siegle J. H. Denman D. J. Bauza M. Barbarits B.. (2017). Fully integrated silicon probes for high-density recording of neural activity. Nature 551, 232–236. 10.1038/nature24636 29120427 PMC5955206

[B27] Kang L. Balasubramanian V. (2019). A geometric attractor mechanism for self-organization of entorhinal grid modules. eLife 8:e46687. 10.7554/eLife.46687 31373556 PMC6776444

[B28] Kang L. Xu B. Morozov D. (2020). State space discovery in spatial representation circuits with persistent cohomology. bioRxiv [Preprint]. 10.1101/2020.10.06.328773 PMC8060447 33897395

[B29] Khalid A. Kim B. S. Chung M. K. Ye J. C. Jeon D. (2014). Tracing the evolution of multi-scale functional networks in a mouse model of depression using persistent brain network homology. Neuroimage 101, 351–363. 10.1016/j.neuroimage.2014.07.040 25064667

[B30] Kropff E. Carmichael J. E. Moser M. B. Moser E. I. (2015). Speed cells in the medial entorhinal cortex. Nature 523, 419–424. 10.1038/nature14622 26176924

[B31] Krupic J. Bauza M. Burton S. Barry C. O'Keefe J. (2015). Grid cell symmetry is shaped by environmental geometry. Nature 518, 232–235. 10.1038/nature14153 25673417 PMC4576734

[B32] Lisman J. E. Jensen O. (2013). The theta-gamma neural code. Neuron 77, 1002–1016. 10.1016/j.neuron.2013.03.007 23522038 PMC3648857

[B33] Mathis A. Herz A. V. M. Stemmler M. (2012). Optimal population codes for space: grid cells outperform place cells. Neural Comput. 24, 2280–2317. 10.1162/NECO_a_00319 22594833

[B34] McInnes L. Healy J. Saul N. Großberger L. (2018). UMAP: uniform manifold approximation and projection. J. Open Source Softw. 3:861. 10.21105/joss.0086133588368

[B35] Mosheiff N. Agmon H. Moriel A. Burak Y. (2017). An efficient coding theory for a dynamic trajectory predicts non-uniform allocation of entorhinal grid cells to modules. PLoS Comput. Biol. 13:e1005597. 10.1371/journal.pcbi.1005597 28628647 PMC5495497

[B36] O'Keefe J. Dostrovsky J. (1971). The hippocampus as a spatial map. Preliminary evidence from unit activity in the freely-moving rat. Brain Res. 34, 171–175. 10.1016/0006-8993(71)90358-1 5124915

[B37] Perea J. A. (2018). Sparse circular coordinates via principal ℤ-Bundles. arXiv arXiv:1510.06629.

[B38] Piangerelli M. Rucco M. Tesei L. Merelli E. (2018). Topological classifier for detecting the emergence of epileptic seizures. BMC Res. Notes 11:392. 10.1186/s13104-018-3482-7 29903043 PMC6003048

[B39] Rybakken E. Baas N. Dunn B. (2019). Decoding of neural data using cohomological feature extraction. Neural Comput. 31, 68–93. 10.1162/neco_a_01150 30462582

[B40] Sanzeni A. Balasubramanian V. Tiana G. Vergassola M. (2016). Complete coverage of space favors modularity of the grid system in the brain. Phys. Rev. E 94:062409. 10.1103/PhysRevE.94.062409 28085304

[B41] Sargolini F. Fyhn M. Hafting T. McNaughton B. L. Witter M. P. Moser M. B.. (2006). Conjunctive representation of position, direction, and velocity in entorhinal cortex. Science 312, 758–762. 10.1126/science.1125572 16675704

[B42] Saxena S. Cunningham J. P. (2019). Towards the neural population doctrine. Curr. Opin. Neurobiol. 55, 103–111. 10.1016/j.conb.2019.02.002 30877963

[B43] Shadlen M. N. Newsome W. T. (1998). The variable discharge of cortical neurons: implications for connectivity, computation, and information coding. J. Neurosci. 18, 3870–3896. 10.1523/JNEUROSCI.18-10-03870.1998 9570816 PMC6793166

[B44] Spreemann G. Dunn B. Botnan M. B. Baas N. A. (2015). Using persistent homology to reveal hidden information in neural data. arXiv [Preprint]. arXiv:1809.09269.

[B45] Sreenivasan S. Fiete I. (2011). Grid cells generate an analog error-correcting code for singularly precise neural computation. Nat. Neurosci. 14, 1330–1337. 10.1038/nn.2901 21909090

[B46] Stemmler M. Mathis A. Herz A. V. M. (2015). Connecting multiple spatial scales to decode the population activity of grid cells. Sci. Adv. 1:e1500816. 10.1126/science.1500816 26824061 PMC4730856

[B47] Stensola H. Stensola T. Solstad T. Frøland K. Moser M. B. Moser E. I. (2012). The entorhinal grid map is discretized. Nature 492, 72–78. 10.1038/nature11649 23222610

[B48] Stensola T. Stensola H. Moser M. B. Moser E. I. (2015). Shearing-induced asymmetry in entorhinal grid cells. Nature 518, 207–212. 10.1038/nature14151 25673414

[B49] Takens F. (1981). “Detecting strange attractors in turbulence,” in Dynamical Systems and Turbulence, Warwick 1980, eds D. Rand and L.-S. Young (Berlin; Heidelberg: Springer Berlin Heidelberg), 366–381. 10.1007/BFb0091924

[B50] Taube J. S. Muller R. U. Ranck J. B. (1990). Head-direction cells recorded from the postsubiculum in freely moving rats. I. Description and quantitative analysis. J. Neurosci. 10, 420–435. 10.1523/JNEUROSCI.10-02-00420.1990 2303851 PMC6570151

[B51] Tenenbaum J. B. Silva V. Langford J. C. (2000). A global geometric framework for nonlinear dimensionality reduction. Science 290, 2319–2323. 10.1126/science.290.5500.2319 11125149

[B52] Wang Y. Ombao H. Chung M. K. (2018). Topological data analysis of single-trial electroencephalographic signals. Ann. Appl. Stat. 12, 1506–1534. 10.1214/17-AOAS1119 30220953 PMC6135261

[B53] Wei X. X. Prentice J. Balasubramanian V. (2015). A principle of economy predicts the functional architecture of grid cells. eLife 4:e08362. 10.7554/eLife.08362 26335200 PMC4616244

[B54] Zhang M. Kalies W. D. Kelso J. A. S. Tognoli E. (2020). Topological portraits of multiscale coordination dynamics. J. Neurosci. Methods 339:108672. 10.1016/j.jneumeth.2020.108672 32151601 PMC7286562

